# A Prospective Evaluation of Tubarial Gland Doses With Acute Dysphagia and Treatment Tolerance in Head and Neck Cancer Patients

**DOI:** 10.7759/cureus.56566

**Published:** 2024-03-20

**Authors:** İpek Pınar Aral, Gonca Altinisik Inan, Binnur Dadak, Fatma Arzu Görtan, Yılmaz Tezcan

**Affiliations:** 1 Radiation Oncology, Ankara Yıldırım Beyazıt University, Ankara, TUR; 2 Radiation Oncology, Ankara Bilkent City Hospital, Ankara, TUR; 3 Department of Nuclear Medicine, Ankara Bilkent City Hospital, Ankara, TUR

**Keywords:** tubarial glands, salivary glands, acute side effect, head and neck cancer, radiotherapy

## Abstract

Background

This study prospectively analyzed the clinical significance of tubarial glands (TGs) doses in head and neck cancer (HNC) patients.

Methods

Patients diagnosed with HNC in Ankara Bilkent City Hospital, Turkey were analyzed. TGs volumes and doses were noted. The patients were evaluated in terms of acute dysphagia (AD) and radiation therapy (RT)-associated xerostomia.

Results

The median volume of the TGs was 3.5(2.1-5.9)cc. No increased standardized uptake values (SUV) were observed in the TGs. There was no significant relationship between TGs values and the third or sixth months of xerostomia after RT. There was a significant relationship between grade ≥2 AD and TGs-D_mean_ (p0.020); TGs-V_25(%) _(p0.007); TGs-V_30(%) _(p0.009); TGs-V_40% _(p0.011); TGs-V_50% _(p0.010), TGs-V_60% _(p0.045). In terms of the risk of grade ≥2 AD, the cut-off value of the TGs-D_mean_ was analyzed for 50 Gy, with 75% sensitivity and 73.3% specificity (p 0.020; AUC 0.746; 95% CI 0.561-0.929). Additionally for grade ≥2 AD, the cut-off value of the TGs-V_25(%) _was analyzed 78 with 81.3% sensitivity and 80.0% specificity (p 0.011; AUC 0.769; 95% CI 0.591-0.947).

Conclusion

A significant correlation was found between TGs doses and AD during RT. TGs-V_25(%) _value showed higher significance. In future studies, the clinical significance of TGs can be studied especially on this value. The relationship between TGs doses and xerostomia should be evaluated with a larger series.

## Introduction

Salivary glands (SGs) are important anatomical structures located within the digestive system, the immune system, articulation, and many other functions. The parotid, submandibular, and sublingual glands are the three major SGs and constitute 90% of saliva production [1]. The SGs are especially important for radiotherapy (RT) of head and neck cancers (HNCs) as a structure that needs to be protected. As a result of radiation-induced damage to SGs, patients may suffer from dry mouth, difficulty swallowing, and an increase in oral infections [2].

Currently, in addition to SGs, "tubarial glands" (TGs) are mentioned as another SGs [3,4]. It is hypothesized that TGs express prostate-specific membrane antigen (PSMA) type 2 transmembrane proteins. It has been reported that high uptake values were observed in PSMA positron emission tomography (PET), such as kidneys, salivary and lacrimal glands, Waldeyer's ring, thyroid, intestine, and pancreas organs expressing the PSMA. Like other SGs, TGs may also show high uptake values in PSMA PET imaging. These high PSMA PET uptake values support the suggestion that TGs should be considered as separate anatomical organs [4,5]. Studies on TGs are mostly performed using PSMA PET and there are a limited number of fluorodeoxyglucose (FDG) PET studies in the literature [3-8]. Matsusaka et al. reported that evaluation of TGs with FDG PET was difficult and TGs uptake was affected by palatine tonsils [6]. Considering all these studies together, the interest in these small structures has increased today.

Although it is expressed as a new organ, TGs have been mentioned before in the literature. The history of TGs is mentioned in detail in Mudry and Jackler's letter in response to Valstar's article [4]. In 1837, the famous anatomist Jean Cruveilhier defined the TGs as "the mucous glands surrounding the Eustachian tube opening, located mainly in the upper part of the pharynx" [4]. The subject has been evaluated throughout the historical process [9]. Another famous anatomist, Jakob Henle, referred to it in 1866 as "the glands of about 2 mm located opposite the choanae, located on the posterior wall of the pharynx" [10]. Autologous Adam Politzer described a network of glands around the tubal orifice in 1878 and later in 1889 [11,12]. In a 1981 article, Tomoda et al. described glandular structures in relation to the nasopharyngeal end of the Eustachian tube. He noted that these had the same appearance as the minor SGs in the oral cavity [9]. In summary, although the discovery of the existence of TGs is not up-to-date, its existence as an organ has not yet been fully accepted. In order for an anatomical structure to gain organ status, it must have its own vascularization. In order to separate from the minor SG, it must show glandular branching. Goldenberg argued that there is not enough data to define these structures as a separate organ. He also emphasized the need for a broader analysis to differentiate it from SG heterotopia [1]. Heterotopic SG tissue can be in the middle ear, larynx, neck, chest wall, sternoclavicular joint, and brain [5,13]. More detailed studies are needed in terms of whether the tubarial area is a heterotopia or a separate organ.

The presence of TGs is important for RT. The reason is that these areas are not protected from HNC irradiation. In the study published by Valstar et al. in 2020, a significant relationship was found between the doses taken by the TGs and grade ≥2 dysphagia and xerostomia. It has been emphasized that the existence of these glands has never been taken into consideration and has not been contoured as a risky organ, and further examination of this issue has been suggested [5]. During HNC irradiation, in addition to other major SGs, contouring of this area as a risky organ has been suggested, but has not yet entered clinical practice [5,9].

The aim of this study was to evaluate the presence/absence of clinical significance of TGs. In this study, the TGs were contoured as a separate SG. The relationship between TG doses and acute dysphagia (AD) and RT-associated xerostomia in patients with HNC was evaluated prospectively.

## Materials and methods

Patients who underwent curative chemoradiotherapy (CRT) with the diagnosis of HNC in the Radiation Oncology Clinic of Ankara Bilkent City, Turkey were analyzed prospectively. Patient file information, electronic system data, patient interviews, and dose volume histograms were used to obtain data. Demographic data, pathology reports, stage, surgical status, details of chemotherapy (CT) and RT, acute side effects, weight loss during RT, dysphagia, and interruption of RT were noted. The study was conducted in accordance with the Helsinki Declaration. The ethical suitability of the study was approved by Ankara Bilkent City Hospital Ethics Committee No. 1 with the number E1-22-2453.

Patients selection

Patients with HNC aged between 18 and 90 years who underwent RT for curative purposes in our clinic were included. Patients who received concomitant CT other than cisplatin had a previous SG disease, had previously received RT, had no pathological diagnosis, and did not receive a direct dose of TG localization such as early-stage larynx were excluded from the study.

Planning and treatment

Before RT, patients were evaluated with FDG PET and dynamic magnetic resonance imaging (MRI). Both imaging and fusion images were used to define the target area of the patients. Discovery RT (GE Healthcare, Chicago, USA) was used for simulation CT. It was performed with a section thickness of 2.5 mm without the use of contrast media. Patients were in the supine position and thermoplastic head and neck masks were made. The TGs were contoured according to the guidelines set in the article by Valstar et al. [10]. TGs were not protected while creating the RT plans. Treatment plans were made by using the Precision planning system (Accuray 1.1, Accuray Inc., Sunnyvale, USA). The patients received RT on the Radixact Treatment Delivery System (Accuray 1.1, Accuray Inc., Sunnyvale, USA).

Patients evaluation

Patients were evaluated weekly by the same clinician. AD grade was noted. RT-associated xerostomia and grade status were evaluated third and six months after RT. Common Toxicity Criteria Adverse Effect (CTCAE) ver 5 (National Institutes of Health (NIH), USA) was used for AD (G1: Symptomatic, able to eat regular diet; G2: Symptomatic and altered eating/swallowing; G3: Severely altered eating/swallowing, tube feeding, total parenteral nutrition (TPN), or hospitalization indicated; G4: Life-threatening consequences, urgent intervention indicated; G5: Death) [14]. 

Xerostomia was assessed according to the Radiation Therapy Oncology Group (RTOG) criteria (G0: None; G1: Slight dryness of mouth, good response on stimulation; G2: Moderate dryness of mouth, poor response on stimulation; G3: Complete dryness of mouth, no response on stimulation; G4: Fibrosis; G5: Death) [15].

Primary and secondary endpoints

The primary aim of the study was to evaluate the relationship between TGs doses and AD and RT-associated xerostomia. Thus, data on the clinical significance of TGs for RT can be obtained. The secondary endpoint of the study was to note the volumetric and dosimetric values of TGs. There is limited data in the literature regarding the volume of this region and the doses it receives.

Statistical analysis

Statistical Package for the Social Sciences (IBM SPSS Statistics for Windows, IBM Corp., Version 26.0, Armonk, NY) was used for statistical analysis. The conformity of the obtained data to the normal distribution was evaluated by visual and analysis methods (Kolmogorov-Smirnov test). Non-parametric tests were used because the data did not fit normally. The relationship between the presence of grade 2 and higher dysphagia of RT and TG doses was analyzed with the non-parametric Mann-Whitney U test. The receiver operating characteristic (ROC) curve of area under the curve (AUC) test was used for the predictive value of TG in terms of RT-related grade 2 and higher dysphagia formation. The statistical significance level was accepted as p <0.05.

## Results

**Figure 1:**
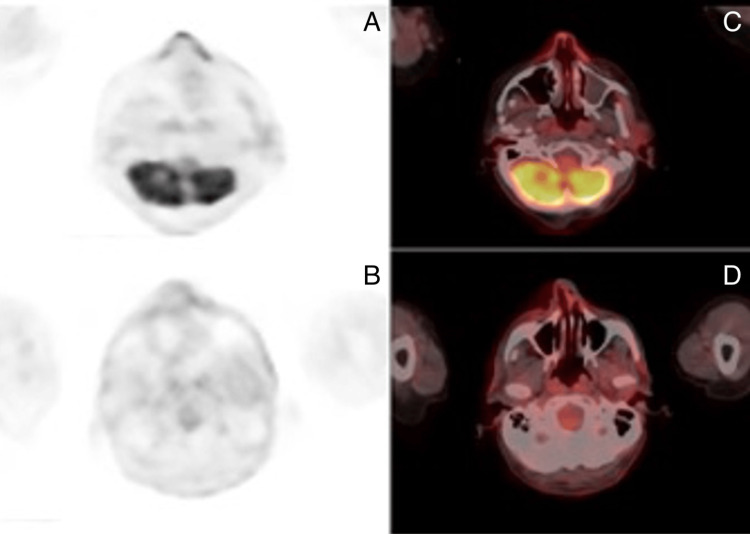
The PET CT image of tubarial glands: MIP (right) (A, B) and axial fused images (left) (C, D) of 18F-FDG PET/CT PET CT: Positron emission tomography-computed tomography, MIP: Maximum intensity projection, FDG: Fluorodeoxyglucose

Results of 31 patients who underwent RT between 04.04.2022 and 12.10.2022 in Ankara City Hospital Radiation Oncology Clinic were evaluated prospectively. The median age of the patients was 60 (range 31-83). At the last control, five patients were dead, and 26 patients were alive. Patients were followed up for at least six months after RT. The median follow-up period was 12 (6-20) months. The median weight was 60 (range 40-109); the median body mass index (BMI) was 23.8 (range 17-42). The majority of the patients (79.6%) were stage 3 and 4. Weight loss was observed in 24 (77.4%) patients during RT. AD was noted in almost all patients (n=30; 96.8%). However, 14 (45.2%) of dysphagia were grade 1. Grade 2 ≥ xerostomia was observed in 13 patients at the third-month follow-up after RT. The six-month evaluation could be performed for 26 patients. In the six-month follow-up, grade ≥2 xerostomia was observed in only two (7.7%) patients. The median value of the right TGs-SUVmean was 1.69 (range 1.07-2.39); the right TGs-SUVmax was 2.61 (range 1.72-3.67). The median value of the left TGs- SUVmean was 1.58 (range 0.99-2.10); the left TGs-SUVmax was 2.47 (range 1.39-3.60) (Figure 1). Patient and treatment characteristics are summarized in Table 1.

**Table 1:** Patients and treatment details BMI: Body mass index; RT: Radiotherapy; CT: Chemotherapy. The data has been represented as N (%). *The six-month evaluation could be performed for 26 patients.

Parameters		
Gender	Female	8 (25.8%)
	Male	23 (74.2%)
Age	Median	60 (31-83)
Weight	Median	60 (40-109)
BMI	Median	23.9 (17-42)
Primary	Nasopharynx	9 (29%)
	Hypopharynx	2 (6.5%)
	Oropharynx	1 (3.2%)
	Larynx	6 (19.4%)
	Oral Cavity	12 (38.7%)
	Maxillary sinus carcinoma	1 (3.2%)
Stage	2	6 (19.4%)
	3	11 (35.5%)
	4	14 (45.2%)
Surgery	No	23 (74.2%)
	Yes	8 (25.8%)
RT Frc Dose	Median	2.12 (2-2.24)
RT Total Dose	Median	70 (55-70)
Neoadjuvant CT	Yes	6 (19.4%)
	No	25 (80.6%)
Concurrent CT	Yes	27 (86.1%)
	No	4 (12.9%)
Weight Loss	Yes	24 (77.4%)
	No	7 (22.6%)
Dysphagia	Yes	30 (96.8%)
	No	1 (3.2%)
	Grade 1	14 (45.2%)
	Grade 2	15 (48.4%)
	Grade 3	1 (3.2%)
RT Interruption	Yes	10 (32.3%)
	No	21 (67.7%)
Xerostomia at third month (after RT)	No	1 (3.2%)
	Yes	30 (96.8%)
	Grade 1	17 (54.8%)
	Grade 2	12 (38.7%)
	Grade 3	1 (3.2%)
Xerostomia at sixth month (after RT)*	No	14 (53.9%)
	Yes	12 (46.1%)
	Grade 1	10 (38.4%)
	Grade 2	2 (7.7%)

**Figure 2:**
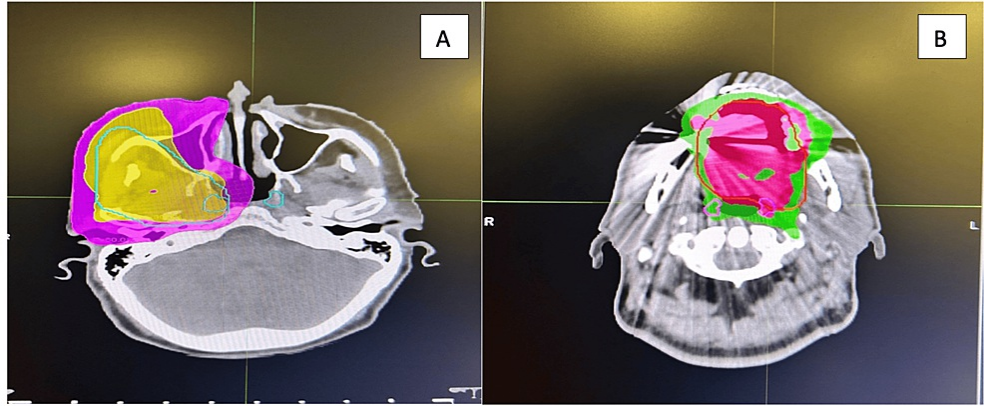
Contour and plan images of tubarial glands: axial images (A, B)

The median volume of the TG was 3.5 (2.1-5.9)cc. TG dose values were as follows: TGs-Dmax median 67.1 (range 12.2-76.5) Gy; TGs-Dmean median 29.5 (range 2.9-73.6) Gy, TGs-V25(%) median 85 (range 0-100); TGs-V30(%) median 83 (range 0-100); TGs V40(%) median 69 (range 0-100); TGs-V50(%) median 56 (range 0-100); TGs-V60(%) median 37 (range 0-100) (Table 2). Volume and dose details of other SGs were summarized in Table 3 and Figure 2.

**Table 2:** Details of TG TGs: Tubarial glands; Dmax: Maximum dose; Dmean: Mean dose; Vx: Volume received X Gy. The data has been represented as median (range).

Parameters	Median (range)
TG Volume	3.5 (2.1-5.9)cc
Right TGs-SUV_mean_	1.69 (1.07-2.39)
Right TGs-SUV_max_	2.61 (1.72- 3.67)
Left TGs-SUV_mean_	1.58 (0.99-2.10)
Left TGs-SUV_max_	2.47 (1.39- 3.60)
TGs-D_max_	67.1 Gy (12.2-76.5)
TGs-D_mean _(Gy)	29.5 (2.9-73.6)
TGs-V_25 (%)_	85 (0-100)
TGs-V_30(%)_	83 (0-100)
TGs-V_40(%)_	69 (0-100)
TGs-V_50(%)_	56 (0-100)
TGs-V_60(%)_	37 (0-100)

**Table 3:** Parotid and submandibular gland details L_PG: Left parotid gland; R_PG: Right parotid gland; L_SMG: Left submandibulary gland; R_SMG: Right submandibulary gland

	Volume (Median) cc	D mean (Median)	D max	V30(%)
L_PG	23.5 (6.6-49.7)	25.3 (2.1-63.2)	64 (23.1-75)	35 (0-54)
R_PG	25.9 (7.3-46.2)	25.7 (2.3-62.7)	65.3 (20.2-75.5)	35 (0-100)
L_SMG	5.6 (0-12.8)	61.2 (0-69.9)	63.9 (0-74)	100 (0-100)
R_SMG	6.1 (0-10.8)	60 (0-73.8)	69.2 (0-76.3)	100 (0-100)

Treatment was interrupted in 10 (32.3%) patients. There was no significant relationship between the interruption of RT and TGs-Dmax (p042); Dmean (p0.19); V25(%) (p0.084); V40% (p0.17); V50% (p0.17); V60% (p0.28). A result close to the limit of significance was obtained only with the V25% value, this value may become significant with increasing the number of patients.

**Figure 3:**
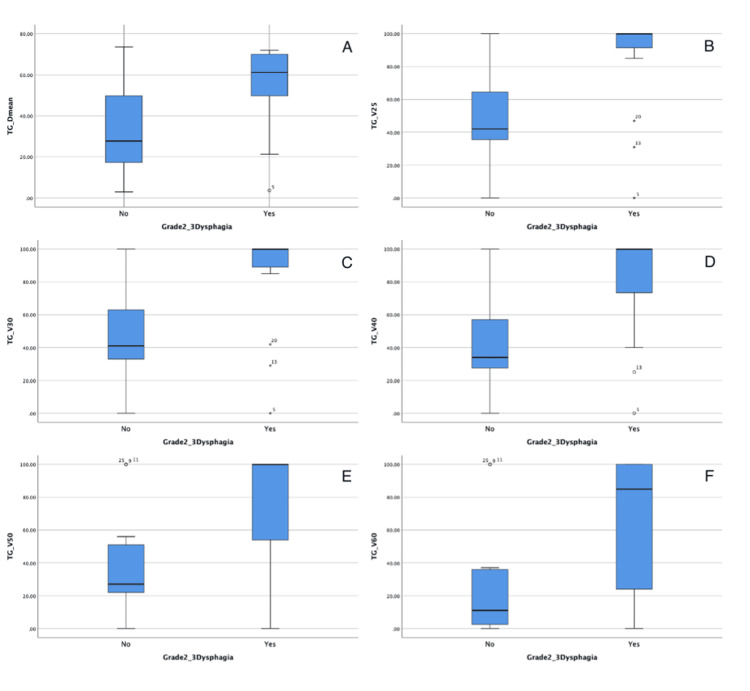
There is a significant relationship between TG doses and grade 2 and higher dysphagia: TG Dmean (A); TG V25 (B); TG V30 (C); TG V40 (D); TG V50 (E); TG V60 (F). All results were statistically significant (p<0.05). TGs: Tubarial glands, Dmean: Mean dose

There was no significant relationship between grade ≥ 2 AD and TGs-Dmax (p0.22; Z:-1227). However, there was a significant relationship between the presence of grade ≥2 AD and TG Dmean (p0.020, Z: -2330); V25(%) (p0.007; Z: -2677); V30(%) (p0.009; Z: -2600); V40% (p0.011; Z: -2546); V50% (p0.010; Z: -2560) V60% (p0.045; -2047) (Table 4) (Figure 3).

**Table 4:** Relationship between salivary gland doses and acute dysphagia TGs: Tubarial glands; Vx: Volume received X Gy. Analyses were performed using the non-parametric Mann-Whitney U test. All results were statistically significant (p<0.05).

	Grade 2-3 (median-range)	Grade 0-1 (median-range)	p	Z
TGs-D_mean _(Gy)	61.2 (3.6-71.9)	27.7 (2.9-73.6)	0.020	-2330
TGs-V_25(%)_	100 (0-100)	42 (0-100)	0.0070	-2677
TGs-V_30(%)_	100 (0-100)	41 (0-100)	0.009	-2600
TGs-V_40(%)_	100 (0-100)	34 (0-100)	0.011	-2546
TGs-V_50(%)_	100 (0-100)	27 (0-100)	0.010	-2560
TGs-V_60(%)_	85 (0-100)	11 (0-100)	0.045	-2047

**Figure 4:**
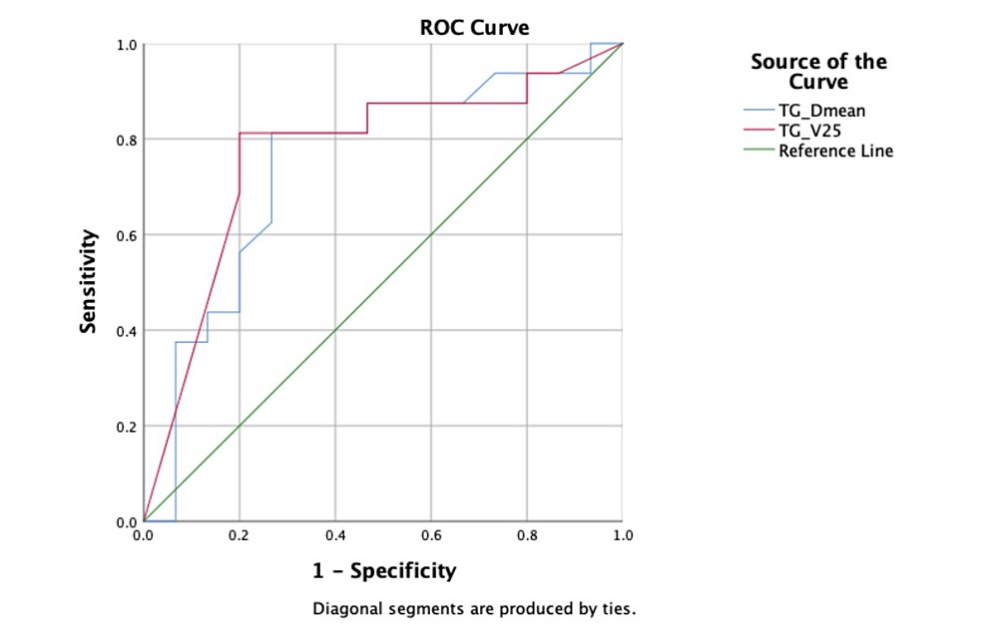
The receiver operating characteristic (ROC) curve analysis image for TG Dmean and TG D25 doses. ROC curve of the area under the curve (AUC) test was used. The results were statistically significant (p<0.05).

The TGs value with the highest significance value was V25(%). ROC curve analysis was performed for TGs-Dmean and TGs-V25(%). In terms of the risk of grade ≥ 2 AD, the cut-off value of the TGs-D mean was analyzed for 50 Gy, with 75% sensitivity and 73.3% specificity (p0.020; AUC 0.746; 95% CI 0.561-0.929). In terms of the risk of grade ≥2 AD, the cut-off value of the TGs-V25(%) was analyzed 78 with 81.3% sensitivity and 80.0% specificity (p0.011; AUC 0.769; 95% CI 0.591-0.947) (Figure 4).

There was no significant correlation between grade ≥2 AD and parotid/submandibular gland doses; L Parotid Dmean (p0.874; Z: -158); L Parotid Dmax (0.353; Z: -929); L Parotid V30(%) (p0.677; Z:-417); R Parotid Dmean (p0.286; Z: -1068); R Parotid Dmax (p0.220; Z: -1226); R Parotid V30(%) (p0.154; Z: -1427); L submand Dmean (p0.267; Z: -1107); L submand Dmax (0.553; Z: -593); L Submand V30(%) (p0.301; Z: -1034); L submand V50(%) (p0569; Z: -580); R submand Dmean (p0.566; Z: -574); R submand Dmax (0.797; Z: -250); L Submand V30(%) (p0.288; Z: -1062); ile L Submand V50(%) (p0.286; Z: -1060).

There was no significant relationship between third-month xerostomia and TGs doses; TGs-Dmax (p0.534); TGs-Dmean (p0.327); TGs-V25(%) (p0.515); TGs-V30(%) (p0.510); TGs-V40% (p0.496); TGs-V50% (p0.448); TGs-V60% (p0,743). Additionally, there was no significant relationship between sixth-month xerostomia and PG doses and submandibulary gland doses; L Parotid Dmax (p0.734); L Parotid V30(%) (p0.457); R Parotid Dmean (p0.280); R Parotid Dmax (p0.207); R parotid V30(%) (p0.221); L submand Dmean (p0.795); L subman Dmax (0.810); L Submand V30(%) (p0.757); L submand V50(%) (p0.925); R submand D mean (p0.737); R submand Dmax (0.550); L Submand V30(%) (p0.684); L subman V50(%) (p0.679). There was only a significant correlation between three-month xerostomia and left PG Dmean dose (p0.039; Z: -2067).

There was no significant relationship between sixth-month xerostomia and TGs doses; TGs-Dmax (p0.213); TGs-Dmean (p0.594); TGs-V25(%) (p0.780); TGs-V30(%) (p0.654); TGs-V40% (p0.712); TGs-V50% (p0.658); TGs-V60% (p0,767). Additionally, there was no significant relationship between sixth-month xerostomia and PG doses and submandibular gland doses; L Parotid Dmean (p0.145); L Parotid Dmax (p0.915); L Parotid V30(%) (p0.284); R Parotid Dmean (p0.644); R Parotid Dmax (p0.213); R parotid V30(%) (p0.593); L submand Dmean (p0.499); L submand Dmax (0.270); L submand V30(%) (p0.123); L submand V50(%) (p0.209); R submand D mean (p0.240); R submand Dmax (0.569); L Submand V30(%) (p0.209); ile L subman V50(%) (p0.280).

## Discussion

In the current study, 31 patients who received RT with the diagnosis of HNC were analyzed prospectively to evaluate the clinical significance of the TGs dose. For TG contouring, Valstar's TG contouring recommendations were used and similar volumetric results were obtained [5]. All of the patients have completed RT; however, treatment was interrupted in approximately one-third of the patients (n=10; 32.3%). There was no relationship between RT interruption and TGs doses, but the statistical relationship with TGs-V25(%) was close to the limit of significance. A significant correlation was found between TGs dose values and grade ≥ 2 AD. There was no statistically significant relationship between TGs doses and xerostomia. Similarly, there was no significant association between other SG doses and xerostomia. When the relationship between clinical results and TGs doses was evaluated in terms of statistical strength, the strongest relationship was with the TG V25 (%) value. We suggest that this value should be taken into consideration for future studies.

In the Valstar study, a significant relationship was found between TG doses and physician-rated xerostomia and physician-rated dysphagia. However, the first patient control of this study was at six months, followed by controls at 12, 18, and 24 months [5]. In this study, unlike Valstar, its relationship with acute side effects during RT has been evaluated. This region is localized in the posterior-superior submucosal area and is adjacent to the constrictor muscles and is likely to be closely associated with AD. Acute side effects, especially AD, during RT seriously reduce the quality of life of patients. It may be necessary to interrupt the treatment in patients who have difficulty in treatment tolerance [16,17]. Interrupting treatment in patients with HNC has a significant adverse effect on oncological outcomes such as local control. The basis of this avoided effect is the clonogenic repopulation observed in the tumoral tissue during the paused period [17,18]. Interruption rates of RT in HNC patients are required in approximately 60-70% of patients [19,20]. In this study, 32.3% of our patients discontinued the treatment. There was no significant relationship between TGs doses and RT interruption. The relationship with TGs- V25(%) values are close to the limit of significance.

There is insufficient data on the pathophysiology of RT-related damage to the upper aerodigestive tract region. After repeated RT fractions, the damage is particularly evident in rapidly proliferating cells. The main causes of damage in healthy tissue are increased oxidative stress, DNA damage, defects in repair mechanisms, and vascular system failure. In addition to submucosal damage, muscle damage, and peripheral nerve injury also contribute to dysphagia [21]. In addition to examination and imaging, these changes can also be detected by patients' complaints [22]. In our study, we observed a significant difference between grade ≥ 2 AD and TGs doses. In addition, we tried to determine threshold values for this region. The relationship between the radiation dose received by this structure located in the posterosuperior part of the pharynx and AD is an expected finding. There are many important anatomical structures in the region, such as mucosal areas and constrictor muscles, and doses to these structures can directly cause AD. Its relationship with grade ≥ 2 AD is not direct proof that the region is an SG. We analyzed the impact of a controversial area on an important issue in terms of radiation oncology.

Today, the debate on the importance of TG as a separate organ has increased after PSMA PET involvement. The involvement of TG structure in other functional imaging was studied by Matsusaka et al. No uptake was observed with (99mTc) pertechnetate SPECT/CT, (18F) FDG PET/CT, and (11C) methionine PET/CT [6]. In the current study, TGs were analyzed using FDG PET. According to our results, the median value of the right TGs-SUVmean was 1.69; the right TGs-SUVmax was 2.61. The median value of the left TGs-SUVmean was 1.58; the left TGs-SUVmax was 2.47. Consistent with the literature, no increased SUV values were observed in the TGs.

If TGs are to be considered SGs, they must be distinguished from the minor SGs. In the article presented by Brandt et al. in 2022, a case of TGs malignancy was presented. In this case report, the authors presented their presentations as minor SG or TGs localization [23]. There are approximately 600-1000 minor SGs in the oral cavity and oropharynx. It is difficult to distinguish TGs from other minor SGs and to define them as a separate organ [23,24]. In the review published by Sainudeen et al. in 2021, it was argued that TGs should be defined as "80 Organs." It was emphasized that research on TGs function and the clinical importance of its preservation in radiotherapy or surgery should be deepened [24]. The size of the minor SGs is usually between 1-5 mm, while Valstar measured the tubular gland size as a mean of 3.9 cm (range 1.0-5.7 cm) [4-6,23]. In our study, the TG volume was median 3.5 (2.1-5.9)cc. We would like to point out that according to Valstar contouring recommendations, TG contouring is not difficult or time-consuming. However, it is not seen in the form of glandular tissue such as submandibular or parotid in CT, it is a more dense structure.

The presence of TGs or identification as a separate organ and its effect on the diseases or side effects observed in the clinic are in interaction. An organ that has not been identified so far may not be considered "clinically significant" if it is not associated with any disease or side effects. Studies investigating its clinical importance are continuing in radiation oncology and ENT clinics. Wu et al., published in 2021, investigated the possible relationship between eustachian tube dysfunction (ETD) and TG. It was emphasized that there is a need for further research on the subject [20]. The clinical significance of TGs as a radiation oncologist can be based on the determination of whether there is a relationship between acute or chronic side effects and whether it is necessary to protect that area. The aim of this study was to investigate the clinical significance of TGs. In this prospective study, in contrast to the parotid and submandibular gland doses, a significant correlation was found between TG doses and grade 2 and higher dysphagia. However, this finding that we have reported indicates the importance of the region. This importance is not direct evidence that the region is a separate major SG. Whether it is associated with xerostomia or not will be more specific in terms of considering TGs as SG. In our third-month and six-month xerostomia evaluation after RT; there was no significant relationship with TG doses. However, the number of patients with grade 2 and higher xerostomia was low. It has been observed that there is a need for a larger series than ours in terms of xerostomia evaluation.

This study has some important limitations. First of all, since the number of patients who developed xerostomia was small, it was not possible to evaluate this issue. Prospective studies with a higher number of patients are required to evaluate this issue more accurately. Additionally, our study was single-center and no randomization was performed. Despite this, our study particularly drew attention to the TGs-V25 dose and has the potential to be a guide for future studies.

## Conclusions

A significant correlation was found between TGs doses and AD during RT. There was no significant relationship between TGs doses and RT interruption, but TGs-V25(%) values were close to the limit of significance. Additional studies are needed for the clinical significance of TGs. Since the number of patients with xerostomia was low, this issue could not be evaluated. For xerostomia, it is necessary to conduct studies on a larger patient population.

## References

[1] Cohen Goldemberg D, Novaes Pinheiro T, Santos-Silva AR, de Melo AC, Leão JC, Fedele S, Porter S (2021). Comments on "The tubarial salivary glands: first description of a potential new organ at risk for head-neck radiotherapy". Radiother Oncol.

[2] Brailo V, Boras VV, Juras DV, Rogulj AA, Brzak BL, Alajbeg I (2017). Oral side effects of head and neck irradiation. Diagnosis and Management of Head and Neck Cancer.

[3] Sakthivel P, Thakar A, Prashanth A (2020). Prostate-specific membrane antigen expression in primary juvenile nasal angiofibroma-a pilot study. Clin Nucl Med.

[4] Mudry A, Jackler RK (2021). Are "tubarial salivary glands" a previously unknown structure?. Radiother Oncol.

[5] Valstar MH, de Bakker BS, Steenbakkers RJ (2021). The tubarial salivary glands: a potential new organ at risk for radiotherapy. Radiother Oncol.

[6] Matsusaka Y, Yamane T, Fukushima K, Seto A, Matsunari I, Kuji I (2021). Can the function of the tubarial glands be evaluated using [(99m)Tc]pertechnetate SPECT/CT, [(18)F]FDG PET/CT, and [(11)C]methionine PET/CT?. EJNMMI Res.

[7] Kumar SA, Meena A, Sood A, Kumar R, Mittal BR (2024). Tubarial salivary glands on PSMA ligands based PET imaging and post (177)Lu PSMA therapy scan: reiterating its importance. Asia Ocean J Nucl Med Biol.

[8] Sample C, Jung N, Rahmim A, Uribe C, Clark H (2022). Development of a CT-based auto-segmentation model for prostate-specific membrane antigen (PSMA) positron emission tomography-delineated tubarial glands. Cureus.

[9] Tomoda K, Morii S, Yamashita T, Kumazawa T (1981). Deviation with increasing age in histologic appearance of submucosal glands in human eustachian tubes. Acta Otolaryngol.

[10] Henle J (1866). Handbook of systematic human anatomy [Article in German]. Braunschweig.

[11] Politzer A (1878). Textbook of otology for practicing physicians and students. 2 volumes [Article in German]. AdeBooks.co.uk.

[12] Politzer A (1889). The anatomical and histological dissection of the human auditory organ [Article in German]. Hansebooks.

[13] Berkovitz BKB, Evans BT, Hopkins C, McHanwell S (2015). Upper aerodigestive tract. Anat Basis Clin Pract Gray’s Anat.

[14] (2017). Common Terminology Criteria for Adverse Events (CTCAE) Version 5.0. https://ctep.cancer.gov/protocoldevelopment/electronic_applications/docs/ctcae_v5_quick_reference_5x7.pdf.

[15] Cox JD, Stetz J, Pajak TF (1995). Toxicity criteria of the Radiation Therapy Oncology Group (RTOG) and the European Organization for Research and Treatment of Cancer (EORTC). Int J Radiat Oncol Biol Phys.

[16] Klimek M, Szostek S (2022). Management of unscheduled treatment interruptions in radiotherapy - mini review. Perceptions Reprod Med.

[17] Yao JJ, Jin YN, Wang SY (2018). The detrimental effects of radiotherapy interruption on local control after concurrent chemoradiotherapy for advanced T-stage nasopharyngeal carcinoma: an observational, prospective analysis. BMC Cancer.

[18] Giddings A (2010). Treatment interruptions in radiation therapy for head-and-neck cancer: rates and causes. J Med Imaging Radiat Sci.

[19] James ND, Williams MV, Summers ET, Jones K, Cottier B (2008). The management of interruptions to radiotherapy in head and neck cancer: an audit of the effectiveness of national guidelines. Clin Oncol (R Coll Radiol).

[20] Wu MJ, Knoll RM, Chari DA, Remenschneider AK, Faquin WC, Kozin ED, Poe DS (2021). Further research needed to understand relationship between tubarial glands and Eustachian tube dysfunction. Otolaryngol Head Neck Surg.

[21] King SN, Dunlap NE, Tennant PA, Pitts T (2016). Pathophysiology of radiation-induced dysphagia in head and neck cancer. Dysphagia.

[22] Pigorsch SU, May C, Kessel KA (2020). MRI- and CT-determined changes of dysphagia/aspiration-related structures (DARS) during and after radiotherapy. PLoS One.

[23] Brandt HH, Baumhoer D, Tetter N (2022). Hyalinizing clear cell salivary gland carcinoma of the epipharynx: a minor salivary/tubarial gland malignancy. Biomed Hub.

[24] Sainudeen S, Sabujan A (2021). Minor salivary glands and ‘tubarial glands’ - anatomy, physiology, and pathology relevant to radiology. J Radiol Clin Imaging.

